# Classifying Reflectance Targets under Ambient Light Conditions Using Passive Spectral Measurements

**DOI:** 10.3390/s20185375

**Published:** 2020-09-19

**Authors:** Ali Hamidisepehr, Michael P. Sama, Joseph S. Dvorak, Ole O. Wendroth, Michael D. Montross

**Affiliations:** 1 Department of Biosystems and Agricultural Engineering, University of Kentucky, Lexington, KY 40546, USA; a.hamidisepehr@uky.edu (A.H.); joe.dvorak@uky.edu (J.S.D.); michael.montross@uky.edu (M.D.M.); 2 Department of Plant and Soil Sciences, University of Kentucky, Lexington, KY 40508, USA; owendroth@uky.edu

**Keywords:** remote sensing, spectroscopy, machine learning, ambient light compensation, reflectance target classification

## Abstract

Collecting remotely sensed spectral data under varying ambient light conditions is challenging. The objective of this study was to test the ability to classify grayscale targets observed by portable spectrometers under varying ambient light conditions. Two sets of spectrometers covering ultraviolet (UV), visible (VIS), and near−infrared (NIR) wavelengths were instrumented using an embedded computer. One set was uncalibrated and used to measure the raw intensity of light reflected from a target. The other set was calibrated and used to measure downwelling irradiance. Three ambient−light compensation methods that successively built upon each other were investigated. The default method used a variable integration time that was determined based on a previous measurement to maximize intensity of the spectral signature (M1). The next method divided the spectral signature by the integration time to normalize the spectrum and reveal relative differences in ambient light intensity (M2). The third method divided the normalized spectrum by the ambient light spectrum on a wavelength basis (M3). Spectral data were classified using a two−step process. First, raw spectral data were preprocessed using a partial least squares (PLS) regression method to compress highly correlated wavelengths and to avoid overfitting. Next, an ensemble of machine learning algorithms was trained, validated, and tested to determine the overall classification accuracy of each algorithm. Results showed that simply maximizing sensitivity led to the best prediction accuracy when classifying known targets. Average prediction accuracy across all spectrometers and compensation methods exceeded 93%.

## 1. Introduction

The ability to sense and quantify spatial variability in parameters of interest within a field is a key component of precision agriculture [1]. In situ and proximal sensing are commonly used for real−time control of agricultural inputs. Remote sensing is suitable for prescriptive management, where measurements are used to build prescription maps that are in turn used to control equipment as it traverses a field. Remote sensing is currently among the most widely studied topics in precision agriculture [2] and the recent advances in small unmanned aircraft systems (sUAS) and miniaturized sensors have provided new tools applied to remote sensing research [3,4]. Remote sensing using sUAS has covered a wide range of applications including sensing biomass and nitrogen status [5], monitoring wheat production [6], and monitoring rangelands [7]. UASs provide a versatile method for remote data collection with a relatively high spatiotemporal resolution when compared to conventional satellite- and ground-based methods [8]. 

Multispectral, thermal, or visible light cameras are most commonly deployed for sUAS-based remote sensing [9,10,11,12,13,14]. Most of the commercially available sensors are designed to work in one or two ranges of wavelengths to reduce sensor cost and data processing complexity. Typically, a small set of narrow−band ranges that are sensitive to one or more field parameters are selected to create an index [15,16]. A ubiquitous index in crop production is the normalized difference vegetation index (NDVI), which typically uses red and near-infrared (NIR) light to estimate crop vigor. While relatively simple to apply, vegetation indices tend to correlate with a myriad of parameters, which makes distinguishing the actual source of variability difficult.

Portable spectrometers are relatively inexpensive tools that can be used to measure a continuous complete spectrum across a wide range of wavelengths. Recent advances in portability and control have led to the ability to mount spectrometers on sUAS platforms [17,18]. In these studies, two identical spectrometers (STS, Ocean Optics) were deployed. One spectrometer was oriented towards the ground and measured the reflectance from a reference white target. The other was mounted on a UAS to measure reflectance from land targets. The ratio of the land target reflectance and the reference white target was considered as compensated reflectance from the land target. Unlike hyperspectral cameras, spectrometers only collect a single spatial measurement representing a circular or elliptical area. Equipment costs and data processing requirements are substantially reduced when using spectrometers versus hyperspectral cameras in instances where spatial resolution is not important.

For lab-based spectrometry, measurements are taken under controlled light conditions, which is an advantage that does not exist for UAS-deployed spectrometers under field condition with frequent changes in ambient light. Experiments that collect spectral measurements are typically conducted during favorable conditions such as full sun around solar noon in order to reduce the effect of ambient light change in measurements and maximize reflectance [19]. Ambient light variability caused by atmospheric conditions reduces the accuracy of measurements derived from spectral data [20]. Thus, spectral measurement systems typically require some form of field calibration to account for ambient light conditions. Calibration of spectral measurement systems is challenging due to the large number of factors that can influence spectral response [21]. Targets with known reflectivity are a vital element in a typical calibration process [22]. The empirical line method is one of the common approaches for calibrating spectral data against variable illumination. In this approach, tarps or panels with known reflectivity are placed in a field during data collection. By finding the relationship between known reflectance values and the raw intensity measurements of the sensor, an equation is obtained and then applied to all measurements [17,18]. The data collection period is limited since changing Sun angle during data acquisition affects the reflectance [23]. Transient cloud cover can also substantially affect the amount of ambient light present over short durations. Another shortfall more specific to hyperspectral imaging is the practical limitation of having tarps or other reference targets in all images, especially when high resolution data is desired or a large area is covered [24].

Devising a method that can keep track of ambient light changes while measuring the raw reflectance from a spectral target using an uncalibrated spectrometer would be useful in precision agriculture research and on-farm applications where ambient light conditions cannot be controlled. By automating this measurement process through concurrent ambient light detection, a compensated reflectance can be obtained for every single wavelength in the spectrum at a low cost and under various ambient light conditions [25,26]. 

Obtained spectrums can be analyzed partially or entirely to estimate different agricultural indices in a field. Nevertheless, calibrating these sensors for various ambient light conditions and avoiding saturation remain a challenge. Ground-based field spectrometers are mostly limited to data collection in a specific period and ambient light condition [27,28,29,30] and use repeated reference measurements from calibration tarps [31]. This process does not scale well to UAS-based applications where large areas are covered and extended time periods with changing ambient light conditions are necessary to acquire measurements.

An alternative to processing a small number of wavelengths into an index is to use the full measured spectrum. Supervised machine learning algorithms are a convenient way to model spectral data. Spectral data are collected from samples with known parameters and used to train the model through a variety of techniques. A subset of data is withheld from training and used to validate or test the model. Different machine learning algorithms have already been used for classification of hyperspectral images [32], weed detection [33], plant disease detection [34], biotic stress detection [35], water quality monitoring [36], human learning [37,38], and many other applications. Several studies focused on developing algorithms and methods for feature selection to reduce the dimensionality of very large datasets [39,40]. Compressing the dataset into a smaller set of components reduces processing time and avoids overfitting the model to the data [41].

Applying machine learning has become less difficult due to advances in computational software, such as MATLAB, that include graphical interfaces for organizing and processing data. Models from one−dimensional spectral data (e.g., intensity vs. wavelength) derived from a few thousand samples can be trained in several minutes using a personal computer. The speed at which models can be trained and validated makes testing a wide range of models feasible. In [42], an ensemble approach to machine learning was used to classify moisture content (MC) of bare soil and wheat stalk residues from spectral data collected in a laboratory controlled experiment. Twenty turn-key models available in MATLAB (R2015b, The Mathworks, Natick, MA, USA) were trained and used to classify MC at seven levels between 3.3% and 30% on a gravimetric basis. Performance varied between 35% and 96% classification accuracy, depending on the model used, and several models had large deviations in performance when classifying soil versus stalk moisture content. Results indicated that choosing a model solely from past performance in literature may not yield optimal performance for a new dataset.

Variability in ambient light conditions and performance of classification methods remains a challenge despite substantial progress in using hyperspectral camera and spectrometer-based remote sensing used to classify agricultural parameters. Many agricultural parameters (e.g., moisture content) are dynamic, which makes collecting comprehensive datasets of spectral data paired with reference measurements expensive. Calibration targets with parameters that do not change under varying ambient conditions are useful first step in testing new sensing and data processing methods before moving on to more complex scenarios.

### Objectives

The application area of this work is remote sensing in precision agriculture using portable spectrometers. Previous work using spectrometers under variable ambient light conditions revealed the need to compensate for ambient light to optimize instrument sensitivity and improve the feasibility of classifying targets from spectral signatures [14,43]. This study aimed to expand upon the previous work by devising methods to update integration time (measurement period) and incorporate calibrated irradiance measurements. Specific objectives included:

1. Fabricate a set of “grayscale” calibration targets and quantify their spectral reflectance relative to a calibration standard.
2. Develop methods for adjusting integration time and incorporating irradiance measurements to automate ambient light compensation.
3. Test the ability of the system to classify different targets under a wide range of ambient light conditions.

The grayscale targets presented in this study were used in lieu of agriculture targets to simplify testing ambient light compensation methods prior to moving on to more complex scenarios.

## 2. Materials and Methods

### 2.1. Instrumentation

**Figure 1:**
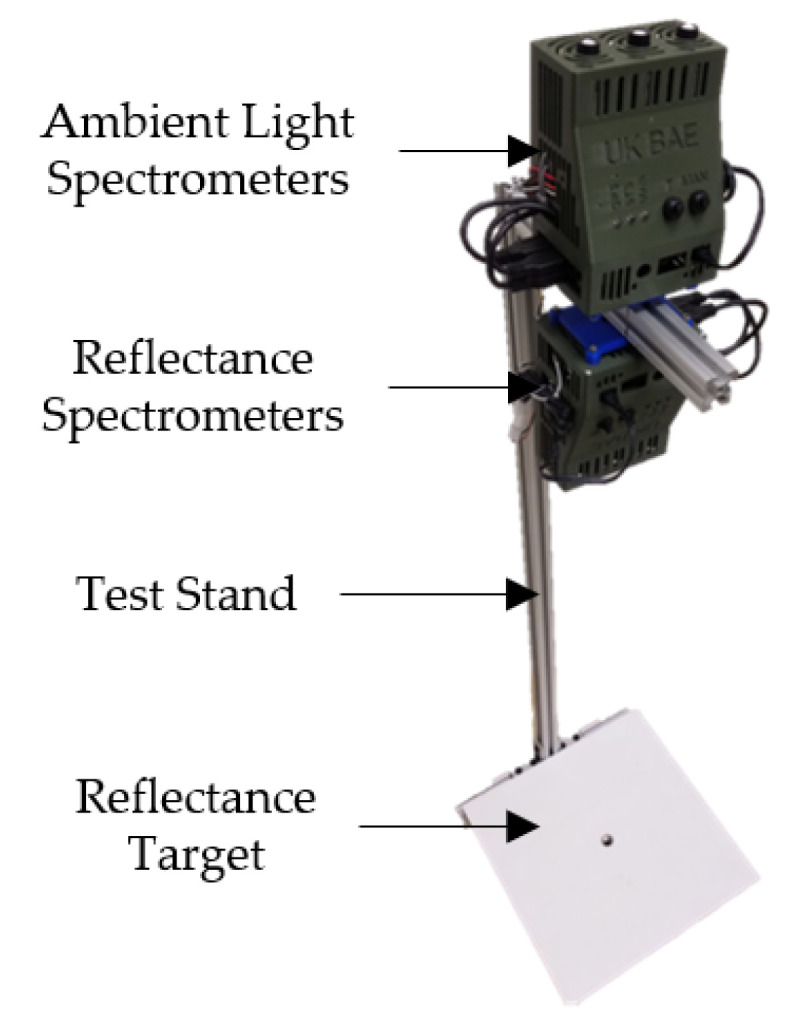
Spectral measurement systems consisting of three ambient light spectrometers and three reflectance spectrometers mounted on a test stand over a reflectance target.

Two spectral measurement systems were deployed for data collection—an ambient light system for collecting downwelling solar irradiance and a reflectance system for collecting upwelling reflectance measurements from targets located underneath the sensors (Figure 1). Each system consisted of three Ocean Optics STS spectrometers in the ultraviolet (UV), visible (VIS), and near−infrared (NIR) ranges; a Raspberry Pi 3 (RPi) embedded computer (Model B V1.2, Raspberry Pi Foundation, Cambridge, United Kingdom); and a custom 3D printed plastic enclosure for mounting each system to a test stand. The test stand aligned the reflectance system 1 m above reflectance targets and positioned the ambient light system directly above the reflectance system.

The UV (STS−UV−L−25−400−SMA, Ocean Optics, Largo, FL, USA), VIS (STS−VIS−L−50−400−SMA, Ocean Optics, Largo, FL, USA), and NIR (STS−NIR−L−25−400−SMA, Ocean Optics, Largo, FL, USA) spectrometers used in the ambient light system were equipped with a direct−attach cosine corrector (CC−3−DA, Ocean Optics, Largo, FL, USA) and factory calibrated to convert raw intensity measurements to units of energy (μJ). The cosine corrector provided a 180° field-of-view (FOV) facing upward and normal to the ground. The UV and NIR spectrometers had an optical resolution of 1.5 nm and the VIS spectrometer had an optical resolution of 3 nm. Spectrometer integration times were fixed at 1000 ms for the UV and NIR spectrometers and 180 ms for the VIS spectrometer. Ambient light spectrometer configurations were selected based on the manufacturer’s recommendation.

The UV (STS−UV−L−100−400−SMA, Ocean Optics, Largo, FL, USA), VIS (STS−VIS−L−100−400−SMA, Ocean Optics, Largo, FL, USA), and NIR (STS−NIR−L−100−400−SMA, Ocean Optics, Largo, FL, USA) spectrometers used in the reflectance system were equipped with a direct−attach collimating lens (74−DA, Ocean Optics, Largo, FL, USA). The 100 μm slit combined with the collimating lens produced an elliptical FOV with a semi−major axis length of 9 cm and a semi-minor axis length of 4 cm. All three spectrometers used in the reflectance system had an optical resolution of 6 nm. Spectrometer integration time varied continuously as described in Section 2.3. Reflectance spectrometer configurations were selected based on the manufacturer’s recommendation. Spectrometer specifications for the ambient light and reflectance systems are summarized in Table 1.

### 2.2. Reflectance Targets

**Figure 2:**
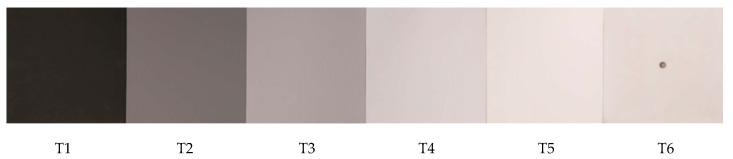
Images of the greyscale reflectance targets used in this study.

Five 0.3 × 0.3 m birch plywood targets painted in varying shades of gray (Glidden Premium Exterior Acrylic Flat Base GL6111 and GL6112, Glidden, Pittsburgh, PA, USA) and one target laminated with a 0.8 mm thick sheet of polytetrafluoroethylene (PTFE), were fabricated as reflectance targets to be placed underneath the spectrometers. Each painted target, labeled T1 through T5 in Figure 2, received two coats of white primer and two coats of paint. The PTFE target, labeled T6, was previously fabricated for a separate study and contained a threaded insert for mounting on a tripod. Targets were offset roughly 12 cm from the center of the reflectance spectral measurement system to ensure that the threaded insert in T6 was not within the FOV when collecting reflectance data.

The relative reflectance of each target was quantified to determine if unique spectral signatures other than changes in average intensity existed, which would oversimplify target classification. A Spectralon calibration standard (WS−1−SL, Ocean Optics, Largo, FL, USA) was used as a benchmark to represent 100% relative reflectance. Actual reflectivity of the Spectralon standard was specified at 99% between 400 and 1500 nm and greater than 96% between 250 and 400 nm. Thus, any non-linearity in reflectivity of the Spectralon calibration target were ignored in this study. A halogen light source (HL−2000−FHSA, Ocean Optics, Largo, FL, USA) was used to illuminate a portion of the target through optical fibers in a backscatter reflectance probe (QR200−12−MIXED, Ocean Optics, Largo, FL, USA). Light reflected from the targets entered separate sets of optical fibers that were fed into two spectrometers (HR4000−7−VIS−NIR & NIRQuest512, Ocean Optics, Largo, FL, USA). The combined spectrometers enveloped the wavelengths observed using the STS UV, VIS, and NIR spectrometers and overlapped at 900 nm. However, spectral response below 400 nm was clipped due to poor sensitivity of the HR4000 spectrometer at shorter wavelengths that resulted in excessive noise in relative reflectance measurements. Nine spectral measurements were taken at uniformly spaced locations across each reflectance target and averaged to quantify relative reflectance response as a function of wavelength.

### 2.3. Reflectance Spectrometer Integration Time

Integration time refers to the period over which a spectrometer detector collects light. Increasing the integration time has the effect of applying a gain to the spectral signal, making weak signals more distinct or unique features more discernable. However, increasing integration time by an excessive amount reduces sampling rate and will eventually lead to saturation in spectral data at one or more wavelengths when the maximum charge that can be stored in an individual pixel has been reached. A saturated measurement is not useful for signal classification. Hence, the optimal scenario is for each measurement to be taken with the maximum integration time that does not result in saturation. In practice, a buffer between the maximum intensity of any wavelength in a spectrum and the saturation level should be maintained to accommodate noise and other uncontrolled processes that could result in saturation.

Reflectance intensity varies when the ambient light condition changes (e.g., due to varying cloud coverage and angle of illumination). A fixed target will produce varying spectral responses using a passive spectrometer if integration time is set constant. A method to automatically update integration time based on the ambient light condition and the spectral response from the previous measurement was devised to optimize spectrometer sensitivity. The process started with setting an initial integration time on each reflectance spectrometer and recording a measurement. A Python script continuously running on a Raspberry Pi then read the most recent measurement. Outliers in the spectral data due to hot pixels (defective pixels that always return a saturated value) were detected and removed. The maximum intensity of the spectrum was determined and compared to the maximum possible intensity without saturation. All STS spectrometers used an identical linear imaging sensor (ELIS−1024, Panavision SVI, Woodland Hills, CA, USA) and intensity at each wavelength was reported as a 14-bit integer value ranging from 0 to 16,383. The units associated with this measurement are referred to herein as counts since they represent the raw output of the analog-to-digital conversion process used to quantify the charge at each pixel of the linear imaging sensor. The maximum desired intensity was set to 12,000 counts to provide a threshold in the event ambient light conditions between measurements were rapidly increasing.

The function used to automatically update integration time is shown in Equation (1). $IT_{k+1}$ represents the integration time for the next measurement in units of milliseconds. $M_{k}$ is the maximum intensity observed in counts for all wavelengths in the current measurement. $M_{max}$ is the maximum desired intensity in counts, set to 12,000 for this study. $IT_{k}$ is the integration time for the current spectral measurement in milliseconds.

(1) $$IT_{k+1}=\frac{M_{k}}{M_{max}}\times IT_{k}$$

The initial integration time prior to the first measurement was set low enough to not result in saturation at any wavelength (UV: 100 ms; VIS: 35 ms; NIR: 100 ms). In the event that a subsequent measurement exhibited saturation, integration time was reset to the initial value and the process of determining the optimal integration time restarted.

### 2.4. Data Collection

Data were collected over five days during September 2017 (9/14/17, 9/15/17, 9/18/17, 9/19,17, and 9/21/17). The ambient light and reflectance systems mounted to the test stand were installed on the roof of the Charles E. Barnhart Building in Lexington, Kentucky (38.027030 N, 84.509641 W). The test stand was oriented to provide an unobstructed line of sight to the Sun so that shadows from the test stand or surrounding objects would not be cast on the targets or ambient light system. Samples were collected in ten-second intervals over a duration of 2 to 3 h on each day. Each sample included three separate measurements that were stored in a tab-delimited text file and averaged to form the sample. The time of measurement and the serial number of the spectrometer were used to define the filenames of text files that stored raw spectral measurements. This filename scheme helped facilitate tracking measurements between the six spectrometers over time. Roughly 3900 pairs of ambient light and reflectance measurements were collected using each spectrometer across all targets.

### 2.5. Compensating for Variable Ambient Light

Ambient light measurements were calibrated from raw intensity in counts to units of energy using a look-up table containing coefficients for different wavelength provided by the spectrometer manufacturer. Equation (2) was used to apply the calibration. $CA_{\lambda \;}$is the calibrated measurement in units of microjoules. $A_{\lambda \;}$is the raw ambient light measurement intensity in counts. $C_{\lambda \;}$is the calibration data in units of counts microjoule^−1^. λ is the individual wavelength.

(2) $$CA_{\lambda \;}=\frac{A_{\lambda \;}}{C_{\lambda \;}}$$

Three successive compensation modes were considered for incorporating the effect of ambient light into reflectance measurements and each mode was evaluated based on the prediction accuracy when classifying targets using machine learning algorithms described in Section 2.7. The automatic integration time method described in Section 2.3 was considered as the first ambient light compensation method (M1). Updating the integration time based on the previous sample optimized the sensitivity of the reflectance spectrometers to the current ambient light conditions. The second ambient light compensation method (M2) divided the resulting intensity value from the reflectance spectrometer by the current integration time ($IT_{k}$) in units of milliseconds to produce intensity relative to integration time. Because all spectra measured using compensation method M1 were anticipated to have similar average intensities, dividing by the integration time would rescale the spectra to have average intensities similar to if a fixed integration time had been used but without sacrificing sensitivity. The third ambient light compensation method (M3) incorporated the calibrated ambient light energy measurements by wavelength as shown in Equation (3). $R_{\lambda \;}$is the compensated reflectance measurement in units of counts ms^−1^ μJ^−1^. $I_{\lambda \;}$is the raw reflectance intensity in counts. The quantity 1500 was the average dark signal present when no light entered the spectrometer and was subtracted from the raw reflectance intensity to remove the offset and provide a zero value when no light was present. By incorporating ambient light energy, compensation method M3 was expected to improve classification accuracy of similar targets when ambient light spectra changed due to uncontrolled external conditions (e.g., cloud coverage, sun angle).

(3) $$R_{\lambda \;}=\frac{I_{\lambda \;}-1,500}{CA_{\lambda \;}\times IT_{k}}$$

### 2.6. Spectral Data Preprocessing

Measurements from each spectrometer covered a distinct range of wavelengths in rough increments of 0.5 nm. The actual spectral ranges for the UV, VIS, and NIR spectrometers were 184−667 nm, 338−825 nm, and 634−1124 nm, respectively. Since data at many of the wavelengths were likely to be highly correlated, partial least squares (PLS) regression was used to reduce the dimensionality of the dataset, solve collinearity issues, and speed up the machine learning classification process. PLS regression reduced the number of input parameters (wavelengths) by representing the full spectrum with a small set of regression components. The optimal number of regression components was obtained using two parameters—the estimated mean squared prediction error when classifying a target and the variance explained in the output variable (target) by the input data (spectral response). The number of regression components in which a high variance in output variable was explained with a low prediction error was considered as the optimal number of input components. The PLS regression method and associated optimization was conducted using MATLAB (R2017a, The Mathworks, Natick, MA, USA).

### 2.7. Target Classification using Machine Learning

The Classification Learner app in MATLAB was used to train 22 different turn−key machine learning algorithms to classify targets based on pre−processed reflectance spectra. An ensemble approach was used here rather than targeting a particular algorithm since the underlying methodology was not of particular interest to this study. The algorithms are generally categorized as decision trees, discriminant analysis, support vector machines (SVM), nearest neighbor classifiers, and ensemble classifiers. Pre-processed spectral data were fed into individual algorithms as a matrix where columns represented regression components (predictors) and rows represented instances of each measurement. The last column (response) was allocated to target codes (T1 through T6). The dataset was randomly subdivided into a training dataset (70%), a validation dataset (15%), and a testing dataset (15%). The training dataset was used to develop the prediction model. The validation dataset was used to determine how well the model has been trained based on the expected output. Model properties, such as classification error and overfitting index were estimated during the validation step to determine if sufficient data had been used to train the model. The testing dataset was used to quantify the classification accuracy of the model by comparing frequency of correct classifications on data not used to train or validate the model. Each model was trained five times with randomly distributed training, validation, and testing data to assess variability when training the model from a finite number of samples. Spectrometer types (UV, VIS, and NIR) and ambient light compensation methods (M1, M2, and M3) were trained independently to determine the best performing combinations with respect to different machine learning algorithms.

### 2.8. Statistical Analysis

Three spectrometers and three compensation modes were considered in this experiment. It was desired to see if there were any significant differences between various types of spectrometers and the ambient light compensation methods in terms of target classification accuracy. The optimal machine learning algorithm was tested for each combination of compensation mode and spectrometer type to determine if significant differences in target classification accuracy existed. The experiment was set up with a factorial design using spectrometer type and ambient light compensation method (3 × 3). The classification accuracy results were subjected to analysis of variance (ANOVA) and a multiple comparison test was conducted using the *anova2* and *multcompare* functions in MATLAB (R2017a), respectively. The *anova2* function tested for significant differences in factors (i.e., spectrometer type and ambient light compensation method) and their interactions. A significance level of 0.05 was used for ANOVA. The *multcompare* function used the output of the *anova2* function to test determine which pairs of factors were significantly different by applying Tukey’s honest significant difference (HSD) procedure. The null hypothesis was that there were no significant differences between spectrometer type and compensation mode with the prediction accuracy of the optimal model.

## 3. Results

### 3.1. Reflectance Target Calibration

**Figure 3:**
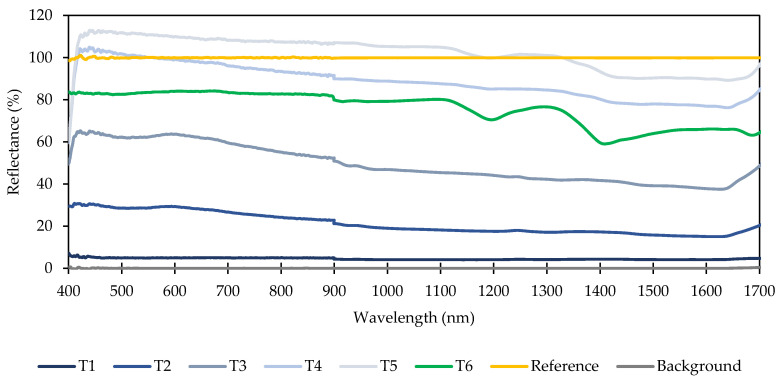
Spectra of reflectance targets (T1 through T6) with lab spectrometers calibrated using the Spectralon calibration standard. UV, VIS, and NIR spectrometers used in the target classification experiment under ambient light conditions only measured light up to 650, 800, and 1100 nm, respectively. Reference and background spectra show reflectance of the calibration standard after calibration with the light source on and off, respectively.

Figure 3 shows the reflectance of each target relative to the Spectralon calibration standard. Reference and background reflectance spectrum are shown as well illustrating the reflectance from the calibration standard (mapped to 100%) and reflectance when the light source was blocked (mapped to 0%), respectively. Unsurprisingly, darker targets reflected less light compared to lighter targets. Painted targets T4 and T5 exhibited relative reflectance in excess of 100% over a range of wavelengths, which indicated that they were “brighter” than the calibration standard. The exact cause of T4 and T5 exceeding 100% reflectance was not known but one potential explanation was the higher sheen of painted targets causing specular reflections. Adjusting the orientation of the backscatter reflectance probe would have likely resolved this issue. A consistent trend across all targets was a general decrease in relative reflectance as wavelength increased until approximately 1650 nm when relative reflectance began to increase. Target T6 (PTFE surface) exhibited the highest variability, particularly in the NIR range. Since the NIR reflectance spectrometer used for ambient light measurements maxed out at 1100 nm, it is unlikely to skew the classification results by providing an easy−to−distinguish target. All targets exhibited noise at short wavelengths, including the reflectance measurements of the calibration standard (reference) after calibration was applied, which was due to the limited sensitivity of the HR4000 spectrometer below roughly 450 nm. The discontinuity at 900 nm was due to the transition between the HR4000 and NIRQuest512 spectrometers.

### 3.2. Ambient Light Measurements

**Figure 4:**
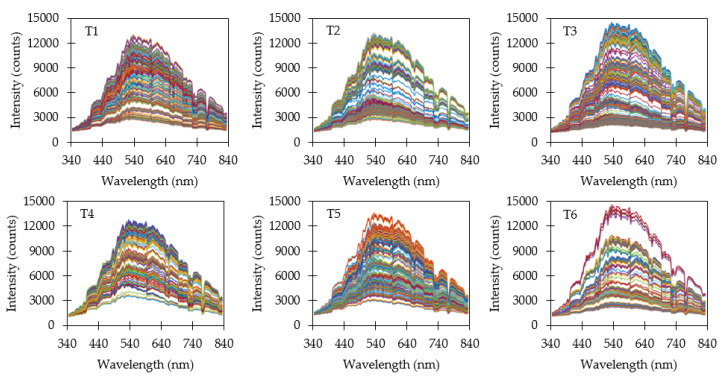
Raw ambient light intensity measurements from the VIS ambient light spectrometer. Each line represents a separate measurement. Line colors are arbitrary and used to help illustrate the number of distinct spectral measurements.

Figure 4 shows all raw ambient light spectra collected by the VIS ambient light spectrometer during the data collection periods for all targets. Each individual line represents a single ambient light measurement and there were typically 820 measurements per target. Line colors were arbitrarily assigned to distinguish separate measurements. Ideally, ambient light conditions would vary uniformly between minima and maxima and be consistent across all targets. Results show reasonable consistency between minima and maxima but there were discrepancies in the distribution of ambient light conditions, particularly when measuring reflectance from target T6. Gaps in the distribution of ambient light were likely due to intermittent cloud coverage. UV and NIR ambient light spectrometers exhibited similar results to the VIS ambient light spectrometer and are not shown.

The maximum difference in raw ambient light intensity occurred around 525 nm and the differences were seven-fold between minima and maxima. This variability directly affects reflectance measurements and illustrates the necessity of compensating reflectance measurements for varying ambient light conditions.

The local variations in intensity were due to the ambient light spectra and the optical characteristics of the spectrometers. Under controlled light conditions, these variations could be removed during calibration similar to what was shown in Figure 3.

### 3.3. Reflectance Measurements

**Figure 5:**
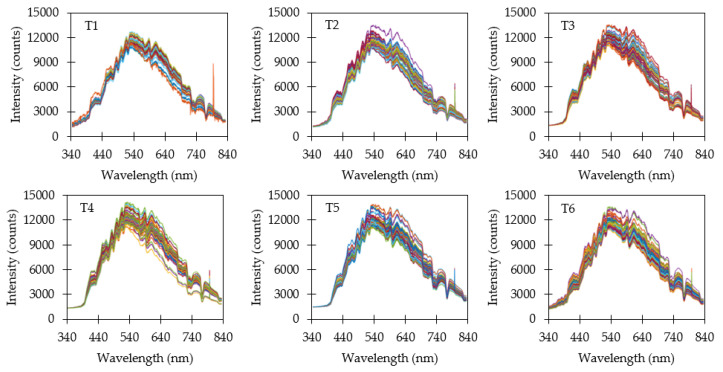
Reflectance measurements using a variable integration time (M1). Saturated and low−intensity measurements due to changing targets were removed from the dataset. Variable integration time caused the reflectance spectra to be tightly grouped near the targeted maximum intensity.

Raw reflectance data when using a variable integration time (M1) are shown in Figure 5. Since integration time was adjusted after each measurement to compensate for varying ambient light, all spectra exhibited similar maximum intensities. Reflectance spectra were filtered to skip saturated measurements and low-intensity spectra. Saturation typically occurred when the target was switched from a dark target to a brighter one. Switching to a brighter target required two subsequent measurements before the integration time stabilized near the optimal value. The first measurement would saturate and cause the integration time to reset to the default value. The second measurement at the default value would result in maximum intensities lower than the target intensity. Conversely, switching from a brighter target to a darker one resulted in low-intensity spectra for at least one subsequent measurement until a sufficient intensity was measured to determine the optimal integration time. 

**Figure 6:**
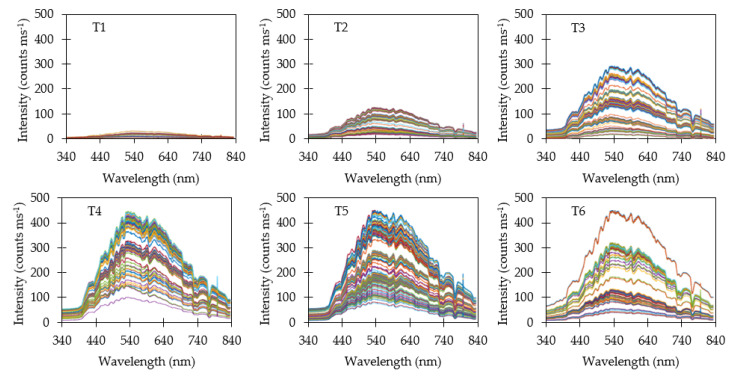
Reflectance measurements divided by integration time (M2). The resulting normalized spectra reveal the average intensity of the target for the three darkest targets (T1, T2 & T3) while the three brightest targets (T4, T5 & T6) still appear similar.

Dividing each raw reflectance measurement by its corresponding integration time (M2) caused several interesting effects on spectra (Figure 6). The darker targets (T1, T2 & T3) were now easy to visually distinguish when plotted on the same scale due to a reduction in the average intensity. The brighter targets (T4, T5 & T6) exhibited similar range of spectra. Variability in average reflectance intensity increased when dividing by integration time due to the variability in ambient light intensity.

**Figure 7:**
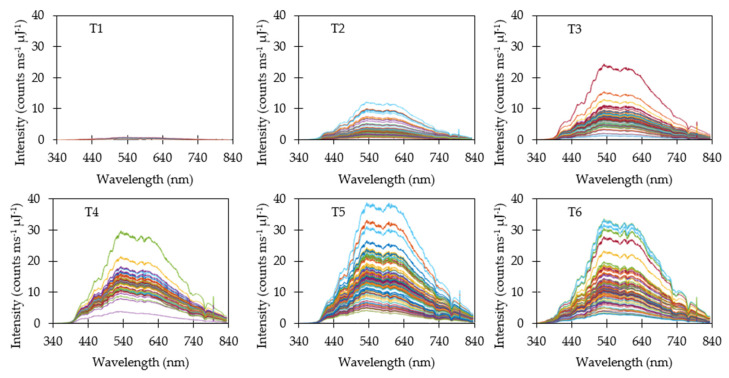
Normalized reflectance measurements divided by ambient light energy (M3).

Dividing yet again by the ambient light energy on a wavelength basis (M3) tended to redistribute and smooth individual spectra (Figure 7). Average intensity was consistent with the reflectance observed relative to the Spectral on standard. A surprising result were the relatively wide distributions of spectral for individual targets. Dividing by integration time and ambient light energy was expected to produce consistent spectra closer to the average spectra, and yet substantial variability remained.

### 3.4. Spectral Data Preprocessing

**Figure 8:**
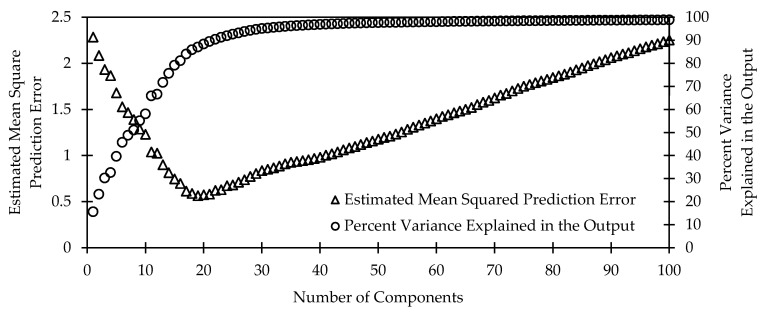
Estimated mean square prediction error and the variance explained in the output versus number of components in a model.

The PLS regression method compressed spectral data consisting of 1024 measurements per spectrometer into a reduced set of components. Figure 8 shows an example of how the number of components affects estimated mean square prediction error and percent variance explained in the output. Estimated mean square prediction error refers to an estimate of how well the model predicts the correct target. Percent variance explained refers to the percentage of variance of the given dataset accounted for by the model. A model with around 20 components produced the lowest estimated mean square prediction error while still accounting for roughly 90% of the variability explained in the output. Increasing the number of components beyond 20 resulted in a linear increase in the estimated mean square prediction error due to overfitting. On the other hand, increasing the number of components resulted in a first−order step response (exponential approach) towards 100% of the variability explained in the output.

### 3.5. Target Classification using Machine Learning

**Figure 9:**
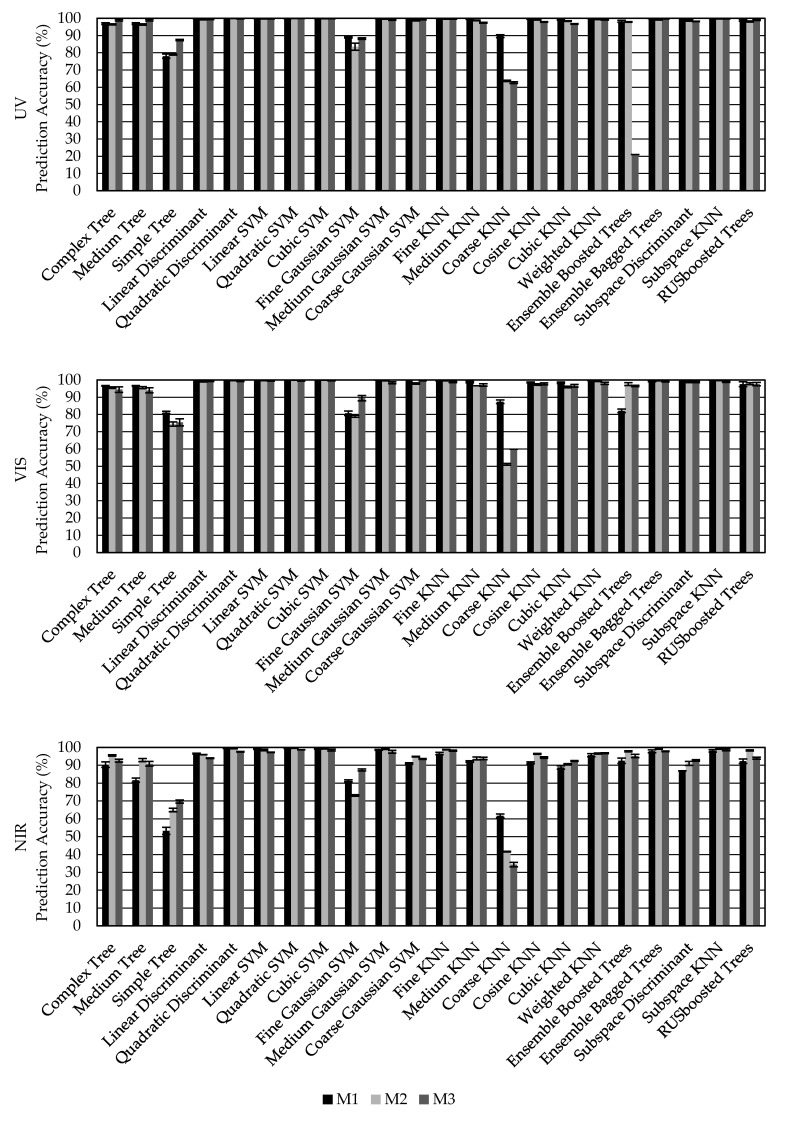
Prediction accuracy for 22 turn-key machine learning algorithms applied to reflectance intensity data collected using UV (top), VIS (middle), and NIR (bottom) spectrometers of six distinct targets and three different methods of ambient light compensation. M1 represents automatic adjustment of the integration time. M2 represents the result of M1 divided by integration time. M3 represents the result of M2 divided by ambient light energy on a wavelength basis. Bars represent average prediction accuracy. Error bars represent ±1 standard deviation.

Figure 9 illustrates the performance of the machine learning algorithms available in the Classification Learning App in MATLAB at the time of this study. Prediction accuracy represents the percent of targets that were correctly identified from the testing data subset. Most machine learning algorithms performed well with prediction accuracies greater than 90% for all spectrometer types and ambient light compensation methods. Discriminant and SVM models tended to produce the most accurate target classifications. The quadratic discriminant performed the best for the UV and VIS spectrometers. The quadratic SVM performed the best for the NIR spectrometer. Variability in results due to the random distribution of training, validation, and testing data was low, as exhibited by the error bars which represent one standard deviation.

Overall, results on which ambient light compensation method produced the best prediction accuracy were mixed. The ambient light compensation method had little effect on prediction accuracy in most instances with the largest deviations occurring when the prediction accuracy was low. The variable integration time method (M1) generally performed the best for the UV and VIS spectrometers and dividing by integration time (M2) performed best for the NIR spectrometer.

Table 2 presents the average prediction accuracy for all targets broken down by reflectance spectrometer, compensation mode, and machine learning algorithm. Several of the machine learning algorithms perfectly classified the targets when using the UV and VIS spectrometers with the variable integration time ambient light compensation method. This result indicates that the targets were likely too easy to distinguish despite the wide range of ambient light conditions. Performance would likely decrease had a larger number of targets or measurements been used. In total, the highest prediction accuracy was obtained using data collected with the VIS spectrometer and applying compensation mode M1 with a model generated using the quadratic discriminant algorithm.

### 3.6. Statistical Analysis

**Figure 10:**
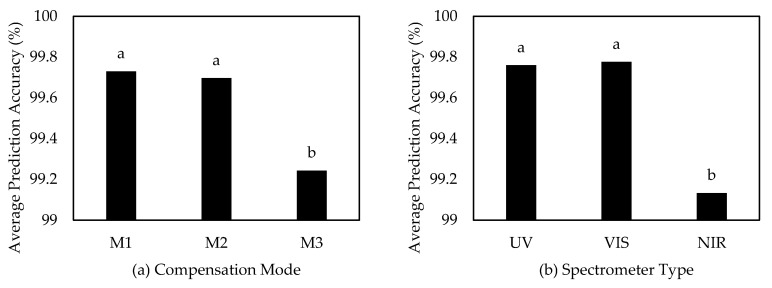
Tukey’s HSD multiple comparison test between different compensation modes (**a**) and spectrometer types (**b**) using the quadratic discriminant algorithm. Bars denoted with different letters are significantly different (α = 0.05).

An analysis of variance was conducted to see if the effect of different compensation modes and the type of spectrometer had significant impact on overall prediction accuracy when using the quadratic discriminant algorithm. Based on Table 3, both treatments had a significant effect on prediction accuracy and the null hypothesis was rejected because of the low p-value (α = 0.05). The results of the Tukey’s HSD multiple comparison test in MATLAB (Figure 10a) showed that the difference between compensation mode M3 and compensation modes M1 and M2 was significant. Compensation mode M1 provided a slightly higher prediction accuracy than compensation mode M2—however, there was no significant difference between these two compensation modes. The NIR spectrometer had a lower overall prediction accuracy on different compensation modes and it was observed from the multiple comparison test that there was a significant difference between NIR and both VIS and UV. No significant effect was observed between UV and VIS spectrometers although prediction accuracy was slightly better when using the VIS spectrometer (Figure 10b).

## 4. Discussion

While not explicitly hypothesis driven, the underlying assumption of this experiment was that dividing normalized spectral measurements by their integration time (M2) and individual wavelengths using ambient light measurements (M3) would improve prediction accuracy when classifying multiple “grayscale” targets across a wide range of ambient light conditions. The results showed that simply optimizing integration time to produce the most sensitive measurement (M1) was the best approach to maximize prediction accuracy.

The results for compensation mode M3 were not surprising given that a second set of calibrated instruments were used to collect the ambient light measurements. The ambient light spectrometers had different optical resolutions from the reflectance spectrometers and, although they report the same wavelengths, incoming light was not distributed across the sensor in the same manner. It might have been more appropriate to simply compute the average ambient light energy from the ambient light spectrometers before applying the normalization rather than by individual wavelength, but the method used in this experiment was chosen to be consistent with existing literature [17,18]. Another potential source of uncertainty is that the integration times of the ambient light spectrometers were fixed while the reflectance spectrometers varied. This resulted in measurements over different periods that may not capture the same variability in ambient light conditions.

The results for compensation mode M2 were not expected given that dividing by the integration time is a scalar operation. A plausible explanation is that the signal that distinguished the targets is not the average intensity but the variability between wavelengths. It is unlikely that the ambient light spectrometers incorrectly applied the desired integration time or incorrectly reported the actual integration time. The small reduction in prediction accuracy may have been due to the rounding that occurred when using integer operations.

While the difference in prediction accuracy between the NIR and the UV/VIS spectrometers was significant, the actual amount was small. Much of this difference can likely be attributed to the targets used. The painted targets did not reflect light uniformly as compared to the Spectralon calibration standard. The most obvious discrepancies between the targets occurred in the UV and VIS ranges, hence the better performance by these spectrometers. A set of “greyscale” calibrated standards with more uniform reflectance would better reveal differences in spectrometer performance. Ultimately, the actual target will define which type of spectrometer should be used for remote sensing. Future work should use more challenging targets, such as crops in a breeding study or soils for moisture analysis, rather than simple “grayscale” targets.

The best performing machine learning methods for classifying targets presented in this study should not be considered optimal for all scenarios. The simplicity of the “greyscale” targets likely masked the true difficulty in classifying parameters in natural targets. Previous work [14] did show that models developed using support vector machines and ensemble bagged trees perform well on agricultural targets, but several of the well-performing models presented here previously failed when using agricultural targets. This emphasizes the importance of not selecting a machine learning model solely based on performance in one domain and further reinforces the need to test ambient light compensation techniques using actual targets for a given application.

## 5. Conclusions

Six “greyscale” reflectance targets were fabricated and benchmarked using a calibration standard. The targets were then used in an outdoor experiment to determine performance differences in ambient light compensation methods when classifying the targets using pairs of ambient light and reflectance spectrometers covering UV, VIS, and NIR wavelengths. Spectral data were collected over five days at varying times during the day to cover a large portion of ambient light conditions. Three successive methods were used to compensate for ambient light variability. The first automatically adjusted the reflectance spectrometer integration time to optimize sensitivity (M1). The second divided the result of the first method by the integration time to normalize the spectrum (M2). The third divided the result of the second method by the ambient light energy on a wavelength basis to directly account for incoming light (M3). The resulting spectra were used to train a series of machine learning algorithms using the Classification Learning app in MATLAB. Most of the algorithms had a prediction accuracy over 90% with an average of 93% across all spectrometer types and compensation methods. The quadratic discriminant model generated from VIS spectrometer data with compensation mode M1 produced the highest prediction accuracy. Statistical analysis revealed that both spectrometer type and compensation mode had a significant effect on the prediction accuracy of targets.

## Figures and Tables

**Table 1:** Ambient light and reflectance spectrometer specifications.

System	Model Number	Nominal Range (nm)	Optics	Optical Resolution (nm) ^1^	Integration Time (ms) ^2^
Ambient Light	STS−UV−L−25−400−SMA	190–650	CC−3−DA	1.5	1000
	STS−VIS−L−50−400−SMA	350–800	CC−3−DA	3.0	180
	STS−NIR−L−25−400−SMA	650–1100	CC−3−DA	1.5	1000
Reflectance	STS−UV−L−100−400−SMA	190–650	74−DA	6.0	Variable
	STS−VIS−L−100−400−SMA	350–800	74−DA	6.0	Variable
	STS−NIR−L−100−400−SMA	650–1100	74−DA	6.0	Variable
	^1^ Optical resolution is set by width of the optical slit. All spectrometers reported 1024 measurements regardless of optical resolution. ^2^ Ambient light integration times were fixed to use the factory calibration when converting raw intensity to energy.				

**Table 2:** Average prediction accuracy for 22 turn-key machine learning algorithms applied to reflectance intensity data collected using UV, VIS, and NIR spectrometers of six distinct targets and three different methods of ambient light compensation. M1 represents automatic adjustment of the integration time. M2 represents the result of M1 divided by integration time. M3 represents the result of M2 divided by ambient light energy on a wavelength basis. Bars represent average prediction accuracy.

Algorithm	M1			M2			M3		
	UV	VIS	NIR	UV	VIS	NIR	UV	VIS	NIR
Complex Tree	96.9	96.6	90.3	96.4	95.6	95.5	98.9	94.4	92.6
Medium Tree	96.9	96.6	81.6	96.4	95.5	92.9	98.9	94.0	90.8
Simple Tree	78.0	81.1	53.2	78.9	74.5	64.9	87.4	75.4	69.6
Linear Discriminant	100	100	96.4	99.3	99.0	95.9	99.4	99.2	93.9
Quadratic Discriminant	100	100	99.5	99.9	99.9	99.4	99.7	99.4	97.5
Linear SVM	99.9	100	99.0	99.6	99.8	98.5	99.7	99.4	97.2
Quadratic SVM	99.9	100	99.5	99.8	99.9	99.7	99.9	99.4	98.6
Cubic SVM	99.9	100	99.3	99.9	99.9	99.4	99.7	99.6	98.5
Fine Gaussian SVM	88.9	80.7	81.2	83.6	78.9	73.0	88.2	89.4	87.3
Medium Gaussian SVM	99.9	100	98.6	99.5	99.5	99.1	99.1	98.4	97.5
Coarse Gaussian SVM	100	100	90.9	98.7	97.9	94.8	99.3	99.5	93.5
Fine SVM	99.9	99.8	96.5	99.6	99.6	98.7	99.5	98.9	98.1
Medium KNN	99.6	98.7	92.1	98.8	96.7	93.8	97.4	97.1	93.8
Coarse KNN	89.7	87.3	61.7	63.7	51.1	41.6	62.7	59.8	34.4
Cosine KNN	99.5	98.5	91.3	99.1	97.3	96.4	97.9	97.7	94.3
Cubic KNN	99.2	98.3	88.7	98.4	95.9	90.5	96.7	96.6	92.4
Weighted KNN	99.8	99.8	95.6	99.5	99.2	96.5	99.0	98.1	96.8
Ensemble Boosted Trees	98.2	86.8	92.5	97.9	97.6	97.8	21.0	96.5	95.2
Ensemble Bagged Trees	99.9	99.2	97.8	99.3	99.6	99.1	99.6	99.0	97.8
Subspace Discriminant	100	100	86.9	98.8	98.8	91.0	98.2	98.8	92.7
Subspace KNN	99.9	100	98.1	99.7	99.6	99.2	99.7	99.0	98.5
RUSboosted Trees	98.8	97.5	92.3	98.1	97.9	98.3	99.1	97.6	93.9

**Table 3:** Significance testing of compensation mode and spectrometer type on overall accuracy of the quadratic discriminant algorithm.

Source	Sum of Squares	Df	Mean Square	F	Prob > F
Compensation mode	4.43	2	2.21	117.31	1.19e−24
Spectrometer type	8.068	2	4.03	213.44	5.13e−33
